# Geographical Disparities in Orthodontic Treatment Demand: 15-Year-Old Vietnamese Population Analyzed by Dentist-Population Ratio

**DOI:** 10.4317/jced.63218

**Published:** 2025-10-01

**Authors:** Hung T Hoang, Nam Cong-Nhat Huynh, Uyen M-N Cao, Chau U Ngo, Quyen L Pham, Khoa D Nguyen

**Affiliations:** 1 Faculty of Dentistry, University of Medicine and Pharmacy at Ho Chi Minh City, Vietnam; 2 Ho Chi Minh City Association of Orthodontists; 3 American Association of Orthodontists

## Abstract

**Background:**

While orthodontists determine the need for treatment, the demand for treatment is a patient’s own decision, based on a questionnaire that addresses their self-perception of their dentition and facial aesthetics. The demand for professional treatment depends on a wide range of factors, such as socioeconomic status, culture, age, and self-perceived appearance, which is sometimes poorly correlated with the normative need for treatment. This study investigated the malocclusion prevalence and orthodontic treatment demand among 15-year-olds in Ho Chi Minh City, considering geographical disparities based on dentist-to-population ratios in three distinct areas: the urban center (6 dentists/10,000 population), para-center (1.6 dentists/10,000 population), and peri-urban areas (0.9 dentists/10,000 population).

**Material and Methods:**

Utilizing multi-stage cluster sampling, 673 children were selected, ensuring representation from each area. Participants completed questionnaires on treatment demand and underwent orthodontic examinations using the Index of Orthodontic Treatment Need (IOTN). Analysis involved conventional statistics and decision tree models.

**Results:**

Results revealed a prevalence of 37% and 46.95% for severe malocclusion, with a noTable demand for treatment (51%), particularly prominent in the urban center area. Decision tree analysis achieved 0.67 accuracy in predicting treatment demand, identifying children from urban centers with DHC-IOTN scores ≥4 as most likely to demand treatment.

**Conclusions:**

Our study reveals geographic disparities in orthodontic demand among 15-year-olds in Ho Chi Minh City, driven by differences in dentist-population ratios. A decision tree model could be effective for clinicians and strategists in determining the most appropriate way to meet the demand for early orthodontic treatment in adolescents across different neighborhoods.

** Key words:**AC-IOTN, DHC-IOTN, malocclusion, orthodontic treatment need and demand, decision tree.

## Introduction

Malocclusion is defined as any deviation from the ideal arrangement of teeth in the jawbone. Although most malocclusions are within accepTable biological variations and do not require treatment, severe cases may have an unfavorable impact not only on dentofacial development and oral function but also on the social and psychological well-being of an individual [1,2]. Orthodontic treatment need refers to the extent of a patient’s malocclusion that needs to be managed by an orthodontist to achieve a normal occlusion, both functionally and aesthetically [3]. Various indices have been proposed to evaluate the severity of malocclusion and the need for orthodontic treatment in the community such as ICON (Index of Complexity, Outcome and Need), DAI ( Dental Aesthetic Index), PAR (Peer Assessment Rating index), OI (Occlusal Index), and NOTI (the Need for Orthodontic Treatment Index) [4-7]. IOTN, developed by Peter H. Brook and William C. Shaw in England (1989), has been widely used to examine the occlusal status and malocclusion prevalence in epidemiological research. This index assesses the functional part known as the dental health component (DHC) and the aesthetic component (AC) [8].

While the need for treatment is determined by orthodontists, the demand for treatment is a patient’s own decision based on a questionnaire, addressing their self-perception of their own dentition and facial aesthetics. The demand for professional treatment depends on a wide range of factors, such as socioeconomic status, culture, age, and self-perceived appearance, which is sometimes poorly correlated with the normative need for treatment [9]. Although multiple studies on this matter have been conducted worldwide; limited data were available in Vietnam because dental health surveys have primarily focused on dental caries and periodontal diseases, while malocclusion and orthodontic treatment needs have been minimally investigated. This study aimed to evaluate the prevalence of malocclusion, the need, and demand for orthodontic treatment among 15-year-old Vietnamese children. We applied Decision Trees as a supervised learning algorithm. This method used a decision tree as a predictive model to go from observations - the prevalence of malocclusion and need for orthodontic treatment (represented in the roots of the tree) to conclusions about the item’s target value - demand for orthodontic treatment (represented in the leaves of the tree).

## Material and Methods

- Study design

This cross-sectional study was conducted from May to September 2019 on 673 subjects aged 15, who were in grade 9 in six junior high schools in Ho Chi Minh City, with their parents’ consent. Inclusion criteria were good health, good cooperation, and no orthodontic treatment.

- Data collection

Data was collected using the multi-stage cluster sampling technique in three different parts of the city: the downtown, urban, and suburban areas. In each area, questionnaires were given to schoolchildren in randomly selected grade 9 classes in 12 secondary schools (4 schools/area) to assess their self-perception and demand for treatment. The subjects were then examined by two trained and calibrated orthodontists using DHC and AC-IOTN in standard daylight and with a DHC ruler, an intraoral mirror, and a lip and cheek retractor. Two standardized photographers took intraoral and extraoral photographs.

In summary, the variables included:

• Area (1: Downtown; 2: urban; 3: sub-urban)

• Gender (1: girl; 2: boy)

• AC-IOTN: Aesthetic Component (from 1 to 10 level) includes a set of ten frontal dental photograhphs of people smiling with their lips relaxed. These photos are scored from 1 to 10, with grade 1 being the most attractive and grade 10 the least attractive.;

• DHC-IOTN: Dental Health Component (from 1 to 5 level), Grade 1 means the occlusion is functionally appropriate and does not need any orthodontic treatment, Grade 5 means the malocclusion is severe and needs correction. DHC assesses five occlusal traits or MOCDO (M for missing teeth, O for overjet, C for cross-bite, D for displacement of contact point, and O for overbite) [10].

• AC-IOTN need3: Need of orthodontic treatment by aesthetic component (1: No need for treatment; 2: Borderline need; 3: Need);

• DHC-IOTN need3: Need for orthodontic treatment by dental health component (1: No need for treatment; 2: Borderline need; 3: Need);

• AC-IOTN need2: Need of orthodontic treatment by aesthetic component (0: no need, 1: need);

• DHC-IOTN need2: Need for orthodontic treatment by dental health component (0: no need, 1: need);

• DEMANDORTHO5: Multileveled demand of orthodontic treatment (Likert scale from 1: no demand to 5: definite demand);

• DEMANDORTHO2: Determined demand of orthodontic treatment (0: no demand, 1: demand).

Statistical analysis

Data were analyzed using JASP software (version 0.18.3, University of Amsterdam). The Chi-square test for qualitative data was applied to compare 3 areas (*p-value* <0.05). We applied Decision Tree Classification using JASP to determine the target Demand of orthodontic treatment based on feature variables. There were 4 models tested: DEMANDORTHO5/ DEMANDORTHO2 targets feature set 1 (area, gender, and components: AC-IOTN, DHC-IOTN)/ feature set 2 (area, gender, and needs: AC-IOTN need3, DHC-IOTN need3, AC-IOTN need2, DHC-IOTN need2).

## Results

- Prevalence of malocclusion and need for orthodontic treatment 

The sample consisted of 673 subjects, including 327 males (48.6%) and 346 females (51.4%) (Supplementary materials Table S1). There was no significant difference between genders and areas. In the suburban area, 38.82% of children had severe malocclusion, followed by urban at 36.24 % and downtown area at 35.5% (Supplementary materials Table S2). The prevalence of severe malocclusion and definite need for orthodontic treatment was 46.95%, with no difference between genders. There was a statistically significant difference in orthodontic treatment need in the different areas in Ho Chi Minh City (*p*< 0.05). The highest percentage of of severe malocclusion was reported in the downtown area (50.5%) (Supplementary materials Materials Table S3).

Prevalence of demand for orthodontic treatment 

Regarding the multilevel demand for orthodontic treatment (DEMANDORTHO5, 5 levels), there were no Level 1 observations (completely no demand) ( Table 1). The prevalence rates of level 2 (likely no demand), level 3 (consideration), level 4 (likely demand), and level 5 (complete demand) were 17.83 %, 46.66 %, 8.92 %, and 26.60 %, respectively. Especially, 40% of children in the downtown area were at level 5 (the highest ratio in the downtown area), while in sub-urban areas, there was 18.04 %. In level 3, sub-urban children accounted for 51.76 % (the highest ratio in these areas).

The determined demand for orthodontic treatment (DEMANDORTHO2: no demand and demand) was recorded for 239 (35.51% of the entire sample). 51% of children in the city center desired to have their teeth corrected, while only 25.88 % expected the treatment in sub-urban areas. There was a statistically significant difference in orthodontic treatment demand with respect to areas in Ho Chi Minh City (*p* = 1.07x10-7) ( Table 2).

- Decision tree analysis targeting multileveled demand for orthodontic treatment 

The decision tree analysis targeting multilevel demand for orthodontic treatment (DEMANDORTHO5) revealed that in the first model (targeting feature set 1: area, gender, and components AC-IOTN, DHC-IOTN), the classification accuracy was 0.66 and which was similar to the second model (targeting feature set 2: area, gender and needs AC-IOTN need3, DHC-IOTN need3, AC-IOTN need2, DHC-IOTN need2) ( Table 3). Other evaluation metrics were found in Supplementary data S4.

**Figure 1:**
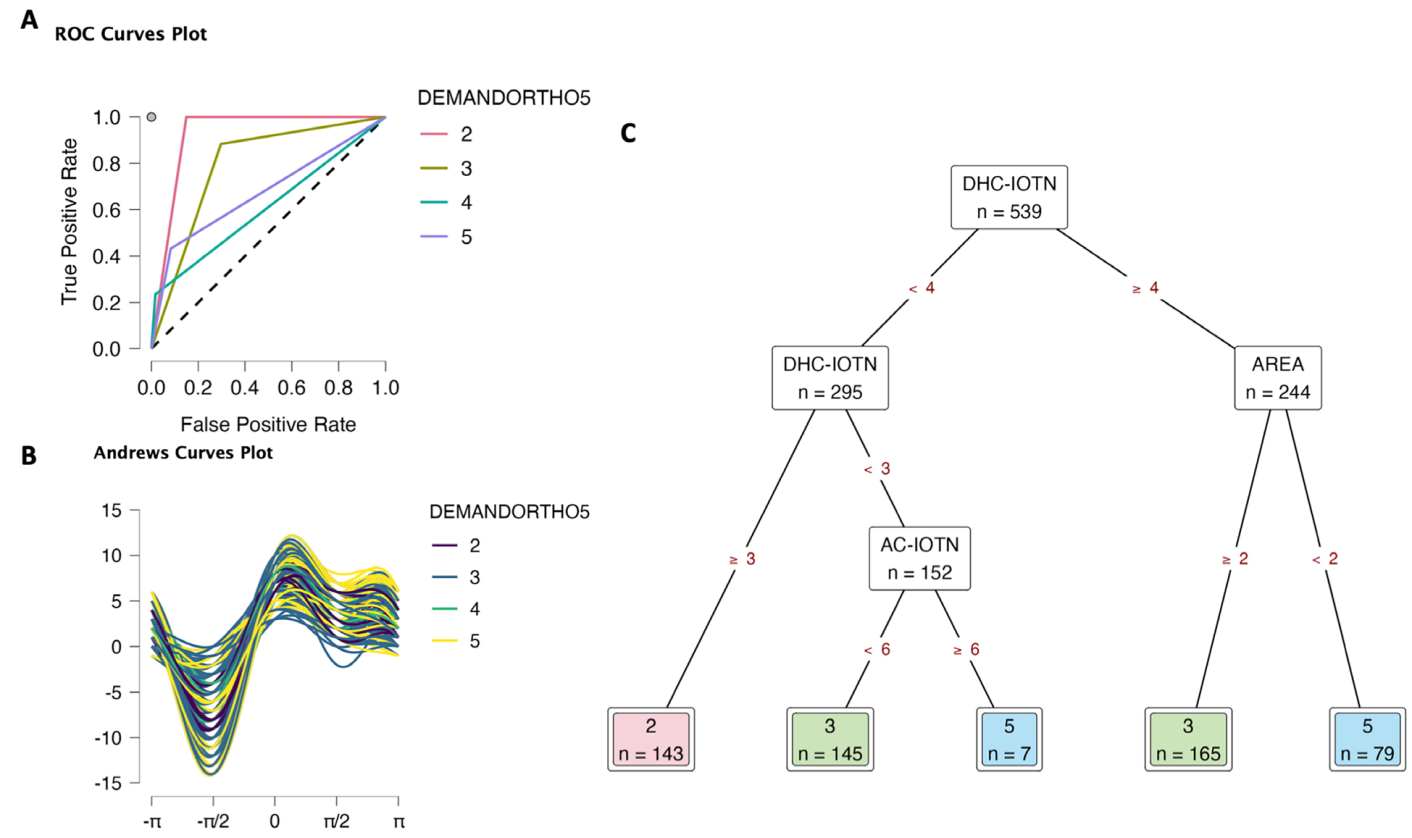
Decision tree classification of orthodontic treatment demand (DEMANDORTHO5 5 levels) based on area (1 Downtown, 2 Urban, 3 Sub-urban), gender (0 Female, 1 Male), aesthetic component (AC-IOTN 10 levels), and dental health component (DHC-IOTN 5 levels). A. ROC curves. B. Andrews curves. C. Decision tree to determine orthodontic treatment demand; only DHC-IOTN, areas, and AC-IOTN took importance in deciding the demand. There was no level 1 observation in this data set; level 4 of DEMANDORTHO5 was predicted randomly.

In the DEMANDORTHO5 (1) model, DHC-IOTN had high relative importance as well as high improvements in the decision tree, followed by AC-IOTN and area ( Table 4, Supplementary data S4), suggesting the importance of the dental health status of children. There was a light role of gender in this model. ROC curves plot for each class predicted against all other classes showed that class 2 had a high true positive rate and a low false positive rate (1 and 0.15, respectively), then class 3 (0.88 and 0.3, respectively), Fig. 1A, Supplementary data S4. On the other hand, class 4 and 5 had a lower true positive rate and a lower false positive rate. The results suggested that the DEMANDORTHO5 (1) model can predict the level 2 and 3 with high accuracy. Andrews curves plot (Fig. 1B) also expressed fairly clear clusters of each class. Finally, the Decision tree (Fig. 1C) visualized the determinants for orthodontic treatment demand. Children with DHC-IOTN ≥ 4 and from the downtown area had definite demand for orthodontic treatment (level 5), but slight demand (level 3) if they were from urban and sub-urban areas. Children with DHC-IOTN <3 and AC-IOTN <6, they also had a slight demand. Children with DHC-IOTN=3 would be level 2 of demand.

**Figure 2:**
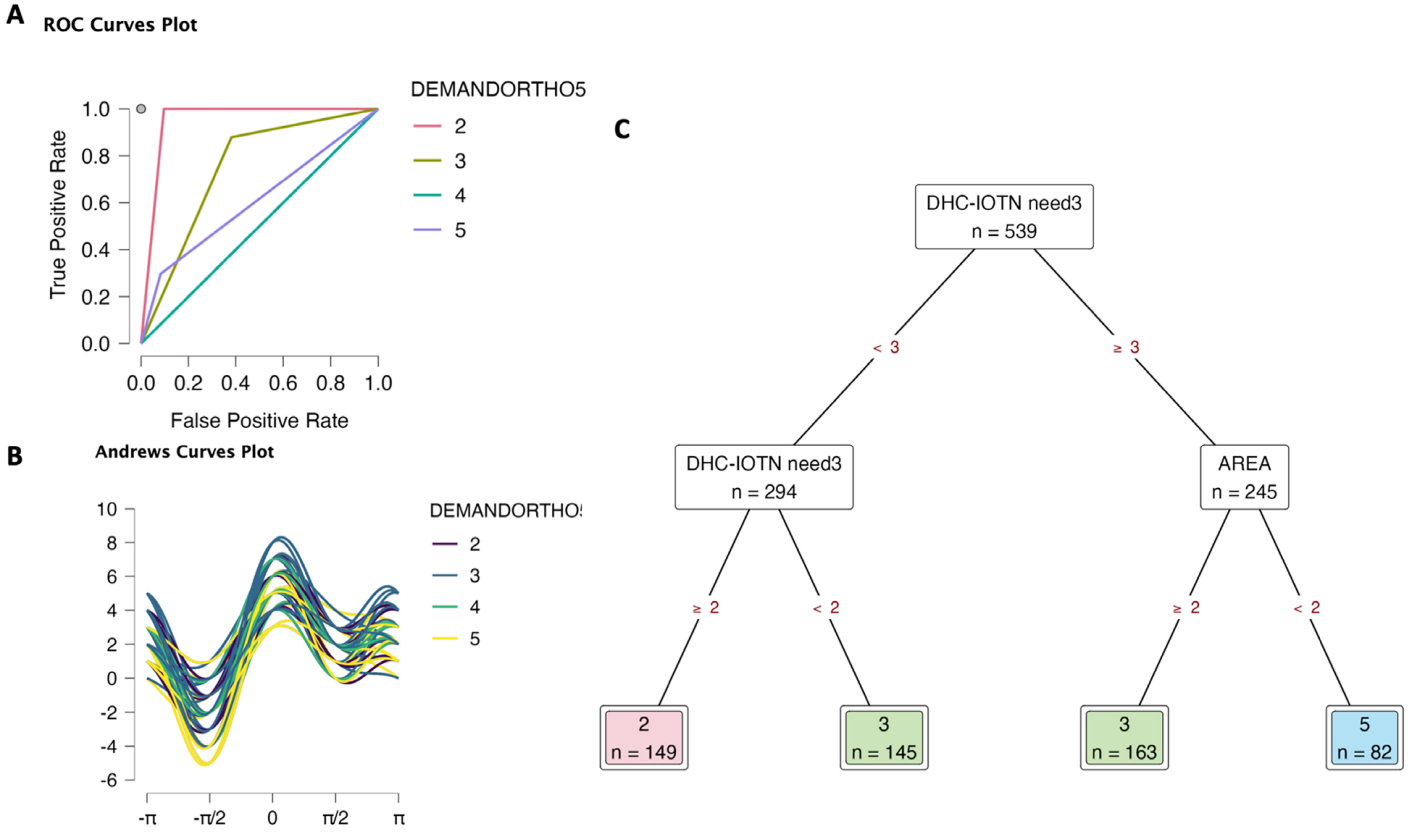
Decision tree classification of orthodontic treatment demand (DEMANDORTHO5: 5 levels) based on area (1 Downtown, 2 Urban, 3 Sub-urban), gender (0 Female, 1 Male), orthodontic treatment need (AC-IOTN need3: 3 levels; DHC-IOTN need3: 3 levels; AC-IOTN need2: 0 No need, 1 Need; DHC-IOTN need2: 0 No need, 1 Need). A. ROC curves. B. Andrews curves. C. Decision tree to determine orthodontic treatment demand; only DHC-IOTN need3 and areas took importance to decide the demand. There was no level 1 observation in this data set; level 4 of DEMANDORTHO5 was predicted randomly.

In DEMANDORTHO5 (2) model, DHC-IOTN need3 contributed the highest to the relative importance (Supplementary data S4). The ROC curves plot showed that the DEMANDORTHO5 (2) model can predict levels 2 and 3 with high accuracy (Fig. 2A, Supplementary data S4). Andrews curves plot (Fig. 2B) was similar to model (1). Decision tree (Fig. 2C) visualized the determination for multileveled orthodontic treatment demand targeting DHC-IOTN need3 and areas. Children with DHC-IOTN need3 = 1 would be level 3 of demand; DHC-IOTN need3 = 2 would be level 2 of demand. Children with DHC-IOTN need3 =3 and from the downtown area would be level 5 for demand (definite demand), but urban and sub-urban had no demand for orthodontic treatment. In the downtown area, children, ones with DHC-IOTN need3 ≥ 3 had slightly higher demand.

Overall, two DEMANDORTHO5 models exhibited accepTable accuracy in predicting multileveled demand for orthodontic treatment, focusing on DHC-IOTN or DHC-IOTN need3 and area features. There was no level 1 observation in this data set; level 4 of DEMANDORTHO5 was predicted randomly.

Decision tree analysis targeting determined demand for orthodontic treatment 

In the decision tree analysis targeting determined demand for orthodontic treatment (DEMANDORTHO2), the first model (targeting feature set 1: area, gender, and components AC-IOTN, DHC-IOTN), the classification accuracy was 0.67, which was and higher than the second model (targeting feature set 2: area, gender, and needs AC-IOTN need3, DHC-IOTN need3, AC-IOTN need2, DHC-IOTN need2), 0.66 (Table 3). Other evaluation metrics could be found in Supplementary data S4.

**Figure 3:**
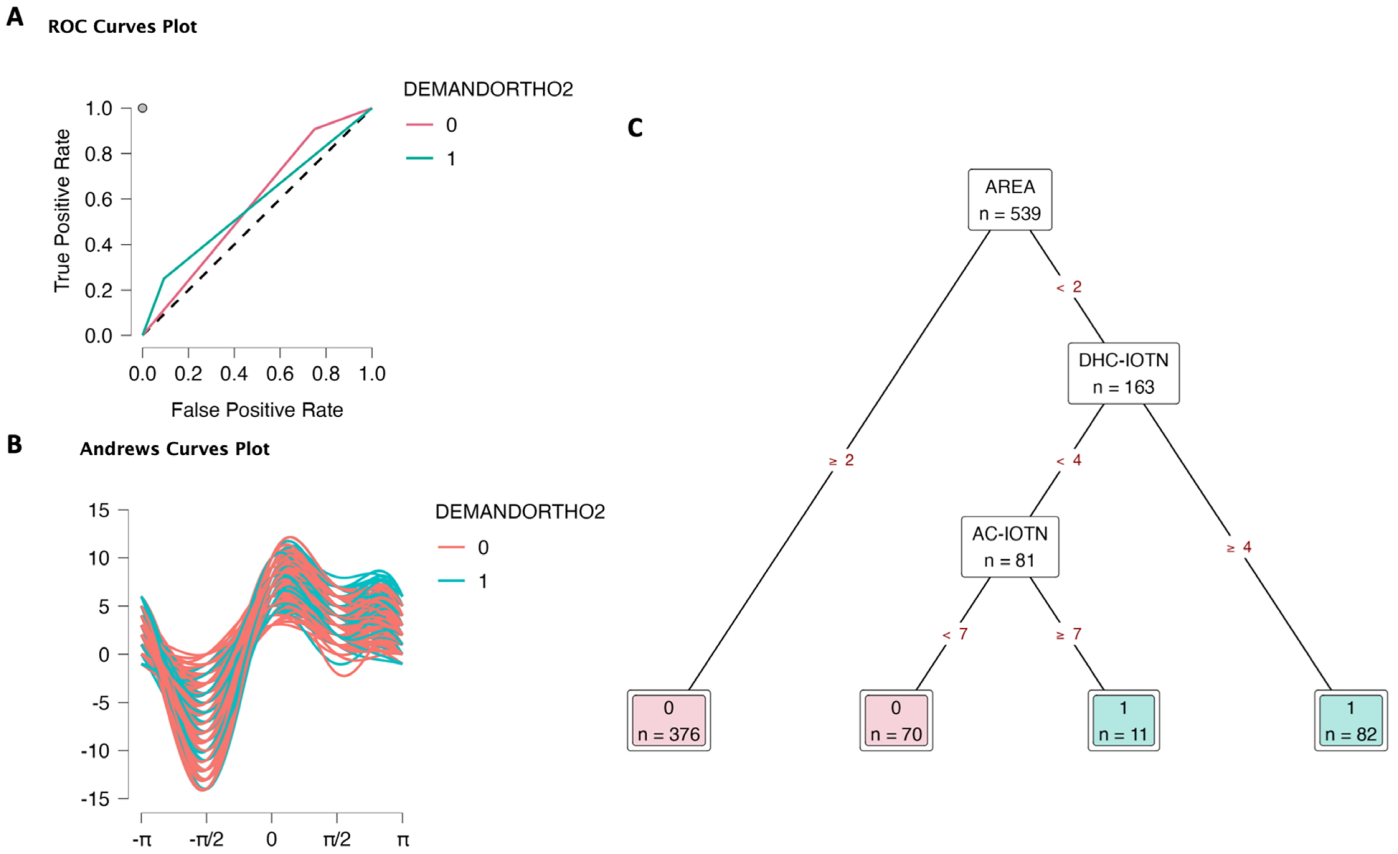
Decision tree classification of orthodontic treatment demand (DEMANDORTHO2: 0 No demand, 1 Demand) based on area (1 Downtown, 2 Urban, 3 Sub-urban), gender (0 Female, 1 Male), aesthetic component (AC-IOTN 10 levels), and dental health component (DHC-IOTN 5 levels). A. ROC curves. B. Andrews curves. C. Decision tree to determine orthodontic treatment demand; only areas, DHC-IOTN, and AC-IOTN took importance in deciding the demand.

In DEMANDORTHO2 (1) model, after area, DHC-IOTN, and then AC-IOTN contributed to the relative importance (Supplementary data S4). ROC curves plot for each class predicted (0: no demand, 1: demand) against all other classes showed that class 0 had a high true positive rate and relative false positive rate (0.91 and 0.75, respectively), Fig. 3A, Supplementary data S4. On the other hand, class 1 had a 0.25 true positive rate and a 0.09 false positive rate. The results suggested that the DEMANDORTHO2 (1) model was accepTable. Andrews curves plot (Fig. 3B) also expressed quite clear clusters of class 0 vs. class 1. Finally, the Decision tree (Fig. 3C) visualized the determination for orthodontic treatment demand. Areas, DHC-IOTN, and AC-IOTN played an important role in deciding this determined demand. Children from urban and suburban areas had no demand for orthodontic treatment. In the downtown area, children with DHC-IOTN ≥ 4 had demand, and those with DHC-IOTN < 4 and AC-IOTN ≥ 7 had demand for orthodontic treatment. Downtown children with DHC-IOTN < 4 and AC-IOTN < 7 had no demand.

**Figure 4:**
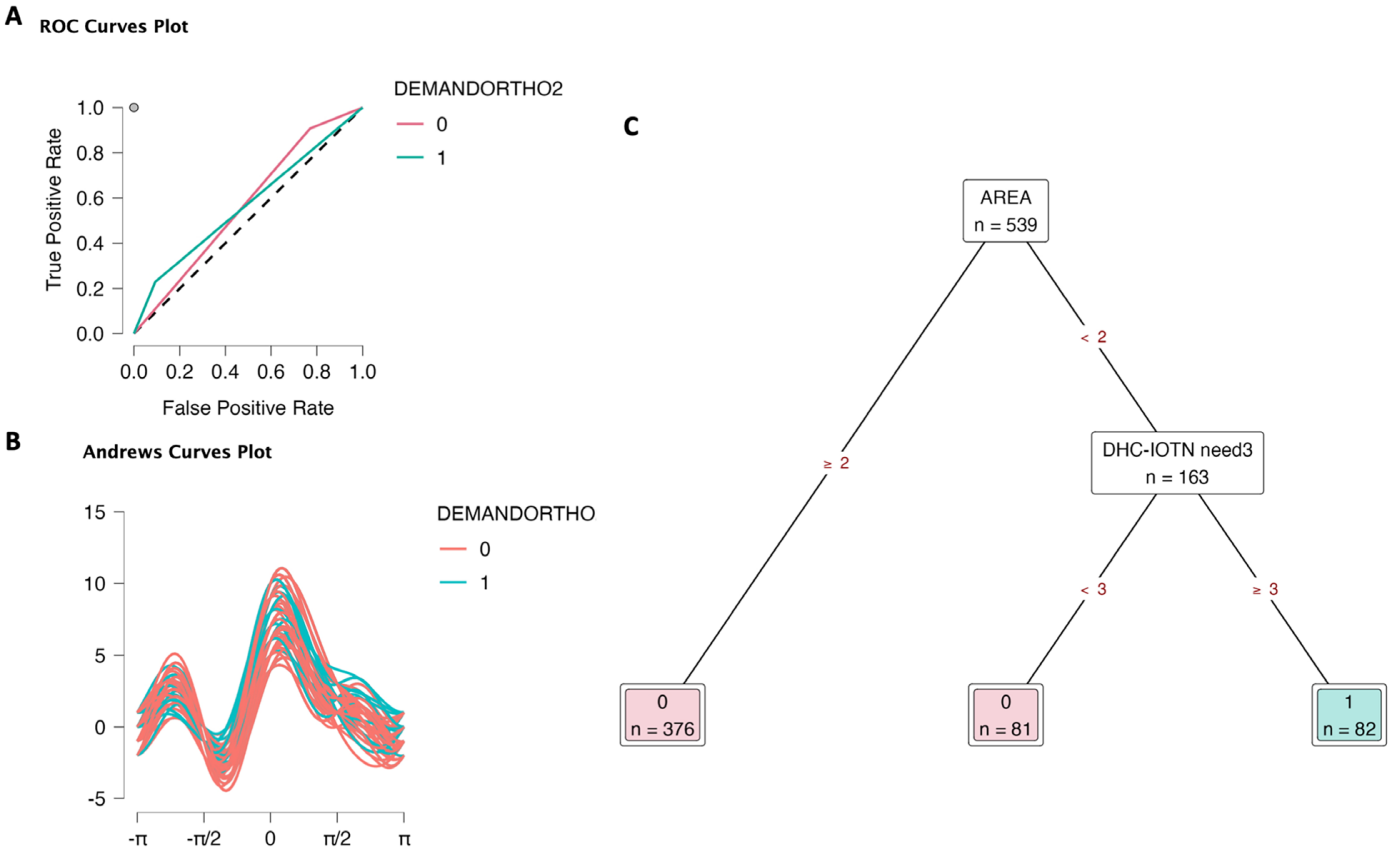
Decision tree classification of orthodontic treatment demand (DEMANDORTHO2: 0 No demand, 1 Demand) based on area (1 Downtown, 2 Urban, 3 Sub-urban), gender (0 Female, 1 Male), orthodontic treatment need (AC-IOTN need3: 3 levels; DHC-IOTN need3: 3 levels; AC-IOTN need2: 0 No need, 1 Need; DHC-IOTN need2: 0 No need, 1 Need). A. ROC curves. B. Andrews curves. C. Decision tree to determine orthodontic treatment demand; only areas and DHC-IOTN need3 took importance in deciding the demand.

Similarly, in the DEMANDORTHO2 (2) model, area and DHC-IOTN need3 contributed to the relative importance (Supplementary data S4). ROC curves plot showed that class 0 had a high true positive rate and relatively high false positive rate (0.91 and 0.77, respectively), Fig. 4A, Supplementary data S4. Additionally, class 1 had 0.23 true positive rate and 0.09 false positive rate. The results were slightly lower than model (1), suggesting that the DEMANDORTHO2 (2) model was accepTable but less accurate than model (1). Andrews curves plot (Fig. 4B) was similar to model (1). Decision tree (Fig. 4C) visualized the determination of orthodontic treatment demand. targeting areas and DHCGROUP. Children from urban and sub-urban areas had no demand for orthodontic treatment. In the downtown area, children with DHC-IOTN need3 ≥ 3 had demand, while children with DHC-IOTN need3 < 3 had no demand.

In general, DEMANDORTHO2 (1) model provided the highest accuracy to predict the determined demand for orthodontic treatment targeting area, DHC-IOTN, and AC-IOTN features.

## Discussion

The IOTN was first used in 2006 by the NHS to classify patients in need of orthodontic treatment as part of primary dental care in a community. Since then, the index has been a useful and reliable guide for specialists to prioritize treatments when resources are limited [10]. In our study, 35.51% of the participants expected to receive orthodontic treatment, which was compatible with 36.3% reported in a study in Sweden, who demanded orthodontic treatment to improve their appearance. However, other studies demonstrated a different percentage of demand in other countries such as the UK, Belgium, and the United States at 41%, 25.7%, and 17.2% respectively [9,11-13]. Treatment demand varied greatly among the three neighborhoods, while nearly half of the subjects in the urban area desired treatment, less than one-third in the rural area wanted treatment. This finding could be due to an appreciation of primary dental healthcare early in life and higher peer pressure among participants living in the urban area. In addition, this study suggested that girls paid more attention to their appearance, evidenced by higher demand for straight teeth, which was in accordance with the observation by Christopherson (2009) [12].

Decision tree analysis has been utilized in studies to predict the demand, targeting the prevalence of malocclusion and the need for orthodontic treatment. This method has been employed to analyze various factors such as demographic data, oral health indicators, socioeconomic status, and treatment accessibility to predict the likelihood of individuals requiring orthodontic treatment. Decision tree models can help identify key predictors and their interactions, providing valuable insights for healthcare planning, resource allocation, and policy-making in orthodontic care [14,15]. In this study, we decided to apply decision tree analysis to determine the demand for orthodontics targeting feature variables among 15-year-old children in Vietnam. Clinicians can conveniently predict the demand from need and area with acceptable accuracy. Interestingly, demand did not consider gender in 15-year-old Vietnamese. Similarly, a path model study by Bayat (2017) predicted orthodontic treatment need and demand, explaining a large proportion of the variance in perceived treatment demand and professionally assessed treatment need [1]. Furthermore, our findings echo those of Ghijselings *et al*. (2014), who reported a low agreement between normative treatment need and self-perceived need in adolescents. Such discrepancies underscore the distinction between clinically assessed need and patient-driven demand in orthodontics [9].

The highest agreement was recorded in the downtown, while the lowest score was observed in the rural neighborhood. It was noticeable that despite the highest percentage of definite need in the rural area, the demand for treatment was the lowest. In fact, area took importance in decision tree models, suggesting the importance of socioeconomic status (downtown vs sub-urban) of the children’s family. It correlated to significant differences in determined orthodontic treatment demand with respect to areas. This finding could be attributed to the lack of early exposure to orthodontic treatment and the socioeconomic status of the participants living on the outskirts of the city. These findings highlight a potential inequity: even when clinical need is high, adolescents in lower-access areas might not translate that need into demand, possibly due to practical barriers or different health perceptions. Our study should be considered as a strategy for oral health promotion in Vietnam, suggesting prevention and early interception in school-based oral healthcare programs as well as training for general dentists/ orthodontists to solve these oral health issues early on for children as a measure of dental public healthcare. Strengthening the training of general dentists and pediatric dentists in early orthodontic assessment and intervention can ensure that children in peripheral areas receive timely guidance or referrals. Such measures would contribute to a more equitable distribution of orthodontic care, so that adolescents across different neighborhoods—regardless of their socioeconomic environment—have the opportunity to receive necessary treatment.

The limitation of the present study included self-reported demand, which may be influenced by subjective bias. The measure of orthodontic treatment demand was based on self-reported data, which is inherently subjective and prone to bias. Adolescents’ self-perception of their orthodontic needs can be influenced by social desirability, peer norms, and individual awareness, potentially leading to over- or underestimation of true demand. The modest accuracy of the model implied that hidden variables or confounders may exist. By employing multiple methods to assess demand, future research can provide a more reliable and nuanced understanding of adolescents’ true desire or intention to seek orthodontic care. Moreover, more complex or ensemble modeling techniques (for instance, random forests or gradient boosting machines) could be explored to see if they yield better accuracy than a single decision tree, as these methods can capture non-linear relationships and interactions between variables that a simple decision tree might miss. Ensuring methodological transparency in model building and validation will be key to translating such predictive models into practical tools. 

In conclusion, the prevalence of severe malocclusion in Ho Chi Minh City was relatively high. Decision tree can be applied to predict orthodontic treatment demand from need and area. More attention should be paid to the sub-urban area community in terms of early and preventive dental healthcare. Dental professionals are now facing new challenges in finding the most appropriate way to satisfy the demand for early orthodontic treatment in adolescents in different neighborhoods.

## Figures and Tables

**Table 1:** Orthodontic treatment demand (DEMANDORTHO5, 5 levels) in 15-year-old schoolchildren in Ho Chi Minh City, no level 1 observation.

Area					
DEMANDORTHO5		Downtown	Urban	Sub-urban	Total
2	n	28.00	35.00	57.00	120.00
	%	14.00%	16.06%	22.35%	17.83%
3	n	70.00	112.00	132.00	314.00
	%	35.00%	51.38%	51.76%	46.66%
4	n	22.00	18.00	20.00	60.00
	%	11.00%	8.26%	7.84%	8.92%
5	n	80.00	53.00	46.00	179.00
	%	40.00%	24.31%	18.04%	26.60%
Total	n	200.00	218.00	255.00	673.00
	%	100.00%	100.00%	100.00%	100.00%

Chi-square test, *p* = 3.27x10-6

**Table 2:** Orthodontic treatment demand (DEMANDORTHO2, 2 levels) in 15-year-old schoolchildren in Ho Chi Minh City.

Area					
DEMANDORTHO2		Downtown	Urban	Sub-urban	Total
No	n	98	147	189	434
	%	49%	67.43%	74.12%	64.49%
Yes	n	102	71	66	239
	%	51%	32.57%	25.88%	35.51%
Total	n	200	218	255	673
	%	100%	100%	100%	100%

Chi-square test, *p* = 1.07x10-7

**Table 3:** Decision Tree Classification of 4 models.

Test	Splits	n(Train)	n(Test)	Test Accuracy
DEMANDORTHO5 (1)	22	539	134	0.66
DEMANDORTHO5 (2)	20	539	134	0.66
DEMANDORTHO2 (1)	12	539	134	0.67
DEMANDORTHO2 (2)	13	539	134	0.66

**Table 4:** Splits in Tree of 4 models.

Models	Variable	Obs. in Split	Split Point	Improvement
DEMANDORTHO5 (1)	DHC-IOTN	539	6.00	35.18
	DHC-IOTN	295	11.00	89.74
	AC-IOTN	152	16.00	3.35
	AREA	244	19.00	10.42
DEMANDORTHO5 (2)	DHC-IOTN need3	539	1.00	38.71
	DHC-IOTN need3	294	14.00	89.83
	AREA	245	19.00	11.68
DEMANDORTHO2 (1)	AREA	539	5.00	12.11
	DHC-IOTN	163	8.00	7.97
	AC-IOTN	81	11.00	3.47
DEMANDORTHO2 (2)	AREA	539	8.00	12.11
	DHC-IOTN need3	163	11.00	7.97

*Note*. For each level of the tree, only the split with the highest improvement in deviance is shown.

## Data Availability

The datasets used and/or analyzed during the current study are available from the corresponding author.
